# Research advances in prevention and treatment of burn wound deepening in early stage

**DOI:** 10.3389/fsurg.2022.1015411

**Published:** 2022-10-21

**Authors:** Meiqi Lu, Jie Zhao, Xiaochuan Wang, Jingjuan Zhang, Fei Shan, Duyin Jiang

**Affiliations:** ^1^ Department of Burns and Plastic Surgery, The Second Hospital, Cheeloo College of Medicine, Shandong University, Jinan, China; ^2^ Department of Emergency Medical Center, The Second Hospital, Cheeloo College of Medicine, Shandong University, Jinan, China

**Keywords:** burns, mechanism, wound progression, mesenchymal stem cells, wound healing

## Abstract

The burn wound is a dynamic living environment that is affected by many factors. It may present a progressive expansion of necrosis into the initially viable zone of stasis within a short time postburn. Therefore, how to salvage of the zone of stasis is of crucial importance in prevention and treatment strategies of burn wound progressive deepening. This review focuses on the cellular basis of tissue injury and the current progress of prevention and treatment strategies of burn wound progressive deepening, in order to provide references for the treatment of burn wounds in the early phase.

## Introduction

Burns wound surface is the source of dynamic local and systemic responses postburn that strongly affect clinical outcome. Meanwhile, the wound changes rapidly in the early phase of burns. In the first fews days, the secondary tissue damage may expand to the initially viable tissues nearby after the primary burn injury both in area and depth (1). Also, a better understanding of the mechanisms that lead to burn wound conversion may lead to more novel therapies that limit burn wound progression in the early stage, and ultimately lead to better healing.

## Pathophysiological changes of early burn wounds

**Figure 1:**
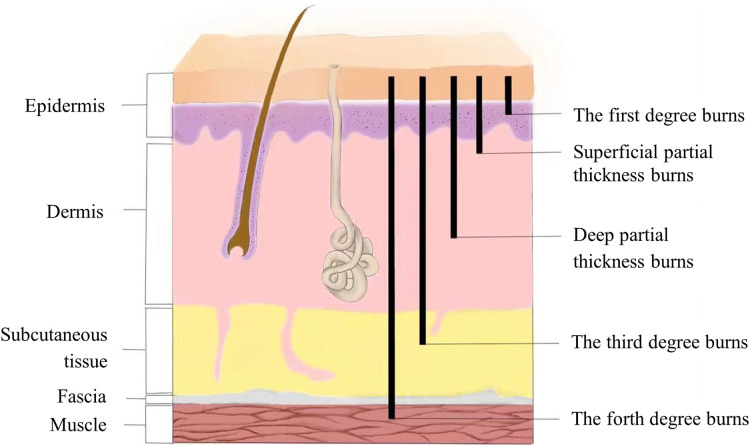
Cutaneous burn classification.

In addition to the immediate coagulation and necrosis of the tissue in the coagulation zone of the skin wound after the burn, the zone of stasis also has a progressive damaging effect in the early stages, especially in the 12–24 h after the injury (2). A range of pathophysiological changes will appear in the zone of stasis: ① Dilation of capillaries and venules, swelling and loose arrangement of vascular endothelial cells, cracks appear between cells, and vascular endothelial cell permeability increases. At the same time, vascular endothelial cells can release a variety of inflammatory mediators and aggravate the inflammatory response. ② During the early phase of burn injury, neutrophils detach from the blood vessels and reach the interstitial space releasing oxygen free radicals, proteolytic enzymes, etc., causing new damage to the tissue and causing the progression of the wound. ③ During the burn, microthrombosis gradually develops due to the accumulation of large numbers of dissolved red blood cells in the lumen (3). The more microthrombosis is formed, the worse the microcirculation state of the wound is, and the degree of tissue necrosis is also deepened, which is one of the reasons for the progressive deepening of the wound after burn (Figure 1).

## Treatment of the wound progression

Progression of burn wounds, or the conversion of superficial burns to deep burns, is characteristic of many burns and leads to worse outcomes. Appropriate treatment of the wound and enhanced protection of the zone of stasis are very important to prevent the deepening of the wound. This section will summarize some of the findings of past studies and highlight the lastest promising developments in various fields.

### Early debridement

There are a lot of inflammatory mediators and endotoxin in eschar and subeschar edema fluid. Improper treatment of early burn wounds makes the wound become the source of infection in burn patients (4). In the early stage of burn, the systemic inflammatory reaction of the patient is not obvious. Debridement in good physical condition can effectively reduce the occurrence of visceral complications and systemic infection after burn, improve the long-term prognosis, and improve the quality of life of the patient. The common methods of debridement used in burn surgery include the sharp debridement and the blunt debridement.

The most common means of removing eschar is escharectomy. The debridement effect of escharectomy is undeniable, but at the same time, excessive removal of healthy tissue is unavoidable. Moreover, hemorrhagic shock is often caused by insufficient hemostasis after extensive excision of eschar. In case of that, there are some precise debridement techniques could be chosen, such as hydrodynamic debridement system (5) and enzymatic debridement (6). These alternative methods of eschar removal that are less traumatic and more selective than escharectomy. Bromelain-based enzymatic debridement and hydrotherapy using pressurized saline flow can effectively remove necrotic tissue with less pain. And the preservation of dermal tissue could reduce surgical burden and improve long-term prognosis (7). Apart of these methods, blunt debridement techniques are promising as well. Dermabrasion and decompression in the initial debridement in the early stage of burns can dredge the blood circulation of the wound, reduce the burn damage, improve the revival of the zone of stasis (8).

### Tissue engineering and stem cell therapy

Tissue engineering has begun to provide cellular therapies for burns and many other tissue injuries in the human body. Among which, stem cell biology has been an important part of the equation when it comes to salvaging the zone of stasis. It is particularly important to preserve the initially unaffected and salvageable stasis area (9) during the treatment process. In recent years, with the development of stem cell technology, its application in wound treatment has also been further developed. Many types of stem cells have been used in clinical management to prevent the delayed necrosis of initially viable tissues surrounding the zone of stasis, such as embryonic stem cell (ESC), somatic stem cell (SSC), induced pluripotent stem cell (iPSC) and mesenchymal stem cell (MSC) (10). In this part, we will focus on the MSCs and describe their usages and functional mechanisms. MSCs can be isolated from different tissues, first from bone marrow, but also from different tissues (11, 12). MSCs represent a type of pluripotent stem cells that can differentiate into various mesenchymal lineages, and are one of the most widely used stem cells in the field of skin injury and repair (13), and play a certain role in protecting the stasis area of burns (14). The mechanisms by which MSCs limit wound deepening and promote wound healing are mainly as follows.

#### Paracrine impact of MSCs

Studies have shown that MSCs can participate in tissue repair through paracrine exosomes to regulate the wound microenvironment (15, 16). Exosomes are nano-sized extracellular vesicles (EVs) secreted by cells, which carry biologically active substances such as nucleic acids, proteins, and lipids, and function in various physiological and pathological processes in the body. For example, mesenchymal stem cell-derived exosomes (MSC-exosomes) are enriched with various miRNAs and proteins that mediate multiple intercellular signaling pathways and reduce inflammation by regulating the levels of various cytokines, including transforming growth factor-β1 (TGF-β1), hepatocyte growth factor (HGF), nitric oxide (NO), and interleukin-4 (IL-4) (17). MSCs can induce regeneration of the epidermal skin barrier by increasing the synthesis of ceramide and dihydroceramides (18). In addition to these functions, MSCs can also support angiogenesis through paracrine influences. Pro-angiogenic factors released by MSCs involve vascular endothelial growth factor (VEGF), placental growth factor (PGF), transforming growth factor-β (TGF-β), platelet derived growth factor (PDGF), angiopoietin-1 (Ang-1), interleukin-6 (IL-6), and monocyte chemotactic protein-1 (MCP-1), which can stimulate the inducing multiple signaling axes to promote therapeutic angiogenesis in wound tissue (19, 20).

#### Multi-directional differentiation of MSCs

Apart from the paracrine pathway, human MSCs are largely thought to differentiate directly into skin cells and participate in wound healing. MSCs are pluripotent stem cells capable of self-renewal and multi-directional differentiation. When it is implanted in the wound, it undergoes spontaneous differentiation under the influence of the wound microenvironment (12). Under suitable conditions, MSCs can differentiate into various types of cells such as nerve cells, vascular endothelial cells, sweat gland cells, and epidermal cells (21–23).

#### Immunomodulatory and anti-inflammatory functions of MSCs

MSCs transplantation can alleviate the inflammatory response in the early stage of burns. After MSCs transplantation in burn wounds, the number of neutrophils infiltrating into the zone of stasis decreased and the activity of myeloperoxidase, which represents tissue neutrophil accumulation, decreased, and pro-inflammatory cytokines such as TNF-α, IL-6, IL-1β and IL-10 expression levels were significantly reduced (14, 24). In addition, MSCs can alleviate burn-induced oxidative stress in the stasis area, which may also be associated with the reduction of inflammatory response (3).

MSCs need to be expanded *in vitro* to perform their regenerative and immunosuppressive functions for various clinical applications. Rombounts (25) found that there was a problem of reduced activity of stem cells after passage: the homing rate of fresh uncultured MSCs after transplantation was 55%–65%, while that of cultured MSCs was 55%–65%. When transplanted after 24 h, the homing rate dropped to 10%. The conventional stem cell transplantation method is to increase the number of stem cells by *in vitro* expansion after the isolated stem cells are obtained. So in stem cell therapy, there is also a trade-off between transplant time and cell viability.

### Measures to reduce local inflammatory response

#### NLRP3 inhibitor

Burn wound infection is one of the important factors leading to early burn wound deepening. The activity of NLRP3 inflammasome is significantly enhanced in macrophages in burn stasis area after scald (26, 27), and inhibiting its activity can improve early burn wound progression and promote wound healing. For example, the NLRP3 inflammasome-specific inhibitor MNS (3,4-Methylenedioxy-β-N) can significantly inhibit the activation of NLRP3 inflammasome and the production of inflammatory factors in burn wounds, and improve the progress of burn wounds. At the same time, increasing the level of autophagy in the wound also inhibited the NLRP3 inflammasome activity in the stasis area. Far-infrared (FIR) irradiation can increase the level of autophagy in the wound, improve the inflammatory infiltration of the wound, and reduce the deepening of the trauma (28).

#### Hyperbaric oxygen therapy (HBO)

HBO improves tissue hypoxia, ischemia-reperfusion injury and reduces pathological inflammation in various clinical settings. It can also shorten the healing time and improve outcomes of patients (29). The skin near II° and III° burns is more hypoxic than normal skin, and the hypoxic tissue around the burn site can be restored to normal oxygen levels by giving oxygen under pressure. HBO can help vasoconstriction to reduce edema, and maintain microcirculation through direct infiltration, enhancing oxygen delivery. HBO also helps inactivate leukocyte adhesion (2) and has a potential broad-spectrum antimicrobial effect (30), so the use of hyperbaric oxygen therapy in the treatment of burns can lead to faster wound healing and reduced morbidity and mortality from complications (31).

### Measures to protect the wound microenvironment

#### Negative pressure wound therapy (NPWT)

NPWT consists of two key techniques: vacuum sealing drainage (VSD) and vacuum assisted closure (VAC). In the early stage of burns, the systemic inflammatory response of patients is not yet obvious. Timely debridement and escharectomy when patients are in good physical condition can effectively reduce the incidence of visceral complications and systemic infections after burns, and improve long-term prognosis (32). Studies have shown that the treatment with VSD after scab grinding in the early stage can increase wound perfusion, shorten wound healing time, reduce redness and swelling of the wound edges, promote drainage of wound secretions, and reduce the incidence of wound infection, effectively prevent the progressive necrosis of the stasis area (33–35). The traditional mode of continuous negative pressure suction mode has shown good results in clinical work, but the continuous negative pressure state can cause the local tissue to adapt to the wound, and the blood perfusion will be insufficient. Under the treatment mode of intermittent negative pressure, the blood flow in the hypoperfusion area near the wound edge is guaranteed, which is conducive to the transport of nutrients and the discharge of metabolites (36), thereby shortening the wound healing time. At present, clinicians are constantly improving the VSD technique in order to achieve better clinical results.

#### Topical dressings

When we treating burn wounds, blister skin should be retained as a biofilm to protect the wound surface, create a moist wound microenvironment, and at the same time reduce the probability of infection, thereby limiting the progressive deepening of the wound surface and promoting wound recovery. For those who cannot retain the blister skin, we also have many artificial dressings to choose from. A variety of wound dressings have been developed, including biosynthetic (skin substitute) dressings, silver-containing dressings, and silicon-coated dressingsgauze, hydrocolloids, and hydrogels (37). The hydrogels are wildly used because of their similarity in structure and composition with natural extracellular matrix. Appropriate pore size helps the hydrogels to retain a moist healing enviroment for wound cell proliferation and angiogen (38). In recent years, researchers have also used xenograft tilapia. The skin covers the burn wound. Tilapia skin has a higher adhesion to wound skin, which can reduce the frequency of dressing changes and the use of analgesics, shorten the time of wound re-epithelialization, and may be a low-cost alternative to accelerate the healing of burn patients and reduce patient pain (39, 40).

### Measures to improve the microcirculation of burn wounds

#### Moistened exposure burn therapy (MEBT)

Moistened exposure burn therapy (MEBT) is a local treatment method that treats burn wounds with moist exposed burn ointment(MEBO) and exposes burn tissue to repair and regeneration in a physiologically moist environment. MEBO applied to the wound surface can effectively improve the microcirculation in the zone of stasis and prevent the progressive necrosis of the tissue nearby (41). The advantages of MEBT are as follows: First, MEBT can effectively improve the microcirculation of the wound, avoiding the formation of a large number of microthrombosis and reducing the degree of ischemia and hypoxia in the zone of stasis. Secondly, MEBT can effectively reduce the capillary permeability and prevent the wound surface from massive extravasation. It relieves wound edema and reduce the possibility of exudative shock in patients after injury (42). Finally, MEBT can effectively reduce the generation of free radicals, inhibit the progressive deepening of the wound caused by lipid peroxidation damage, and play a role in the tissue protection in the stasis area.

#### Cold therapy

Appropriate cold therapy can reduce local thermal damage, decrease edema, inhibit the release of oxygen free radicals, reduce inflammation, and effectively improve local wound microcirculation. Treatment with moderate hypothermia at 31°C–33°C for 4 h, starting from 2 h after the burn resulted in a 23% reduction in burn depth compared to the control group at 24 h postburn. Simultaneous hypothermia-induced upregulation of skin protective genes such as CCL4, CCL6, and CXCL13 and downregulation of deleterious tissue remodeling genes such as MMP-9 may contribute to improved burn depth progression (43). However, perfusion in the zone of stasis may be further affected by vasoconstriction caused by supercooling, leading to further wound deterioration. The perfusion of the zone of stasis was somewhat improved under warm water (37°C), and the tissue viability was enhanced (44).

#### Early anticoagulation therapy

The blood in the early stage of burn is hypercoagulable, leading to stasis of microcirculation and microthrombosis, which is one of the important mechanisms for the development of stasis zone into coagulation zone (45). In recent years, anticoagulant and thrombolytic drugs have emerged to improve microcirculation thrombosis and vascular occlusion. For example, the well-known human erythropoietin (EPO) is a multifunctional cytoprotective cytokine in addition to promoting erythropoiesis, with anti-apoptotic, anti-inflammatory and immunomodulatory properties. Bohr et al. (46, 47) showed that systemic administration of the EPO derivative helical β-surface peptide (ARA290) within 24 h postburn prevents secondary microvascular thrombosis and inflammatory response in skin burns, and improves local microcirculation, while reducing the cellular stress response mediated by inflammatory factors such as TNF-α, thereby reducing the further deepening of the depth and area of burn wounds. Secondly, increased plasma endothelin levels following burns lead to thrombosis and occlusion of vessels in the dermis and vascular responses in the adjacent uninjured dermis. Previous studies have demonstrated that non-selective endothelin receptor antagonists [TAK-044 (48), PD145065 (49), etc.] can improve local microcirculation.

## Conclusion and prospect

Burns are one of the most common and devastating forms of trauma. In the clinical environment, early debridement, early wound coverage, and the application of various therapies to promote healing can improve the progressive deepening and aggravation of early burn wounds, avoid the pain caused by large-scale surgical operations to a certain extent, and improve long-term outcomes. Given the complexity and diversity of the principles of each treatment, the categories mentioned above do not encompass the full range of functions of the measures mentioned. Combining multiple therapies may lead to better clinical benefits. We can also see that many therapies have their shortcomings, such as the issue of stem cell homing rates, and many drugs are still in clinical trials. At the same time, there are few studies on exosomes derived from mesenchymal stem cells. Their mechanisms of action overlap and need further research study.
